# Dental Pulp Stem Cell Heterogeneity: Finding Superior Quality “Needles” in a Dental Pulpal “Haystack” for Regenerative Medicine-Based Applications

**DOI:** 10.1155/2022/9127074

**Published:** 2022-01-04

**Authors:** Zi Y. Kok, Nadia Y. A. Alaidaroos, Amr Alraies, John S. Colombo, Lindsay C. Davies, Rachel J. Waddington, Alastair J. Sloan, Ryan Moseley

**Affiliations:** ^1^Regenerative Biology Group, Oral and Biomedical Sciences, School of Dentistry, Cardiff Institute of Tissue Engineering and Repair (CITER), College of Biomedical and Life Sciences, Cardiff University, Cardiff, UK; ^2^Tissue Microenvironment Group, Division of Cancer and Genetics, School of Medicine, College of Biomedical and Life Sciences, Cardiff University, Cardiff, UK; ^3^School of Dental Medicine, University of Nevada Las Vegas, Las Vegas, Nevada, USA; ^4^Department of Laboratory Medicine, Karolinska Institutet, Huddinge, Sweden; ^5^Melbourne Dental School, Faculty of Medicine, Dentistry and Health Sciences, The University of Melbourne, Melbourne, Australia

## Abstract

Human dental pulp stem/stromal cells (hDPSCs) derived from the permanent secondary dentition are recognised to possess certain advantageous traits, which support their potential use as a viable source of mesenchymal stem/stromal cells (MSCs) for regenerative medicine-based applications. However, the well-established heterogeneous nature of hDPSC subpopulations, coupled with their limited numbers within dental pulp tissues, has impeded our understanding of hDPSC biology and the translation of sufficient quantities of these cells from laboratory research, through successful therapy development and clinical applications. This article reviews our current understanding of hDPSC biology and the evidence underpinning the molecular basis of their heterogeneity, which may be exploited to distinguish individual subpopulations with specific or superior characteristics for regenerative medicine applications. Pertinent unanswered questions which still remain, regarding the developmental origins, hierarchical organisation, and stem cell niche locations of hDPSC subpopulations and their roles in hDPSC heterogeneity and functions, will further be explored. Ultimately, a greater understanding of how key features, such as specific cell surface, senescence and other relevant genes, and protein and metabolic markers, delineate between hDPSC subpopulations with contrasting stemness, proliferative, multipotency, immunomodulatory, anti-inflammatory, and other relevant properties is required. Such knowledge advancements will undoubtedly lead to the development of novel screening, isolation, and purification strategies, permitting the routine and effective identification, enrichment, and expansion of more desirable hDPSC subpopulations for regenerative medicine-based applications. Furthermore, such innovative measures could lead to improved cell expansion, manufacture, and banking procedures, thereby supporting the translational development of hDPSC-based therapies in the future.

## 1. Introduction

The dental pulp tissues of postnatal human teeth are now well-established to harbour a unique and varied source of mesenchymal stem/stromal cells (MSCs). Due to the essential roles that MSCs play during tissue development and in mediating reparative responses within the dentine-pulp complex [1], there is a strongly held belief that the dental pulp offers a potentially viable source of MSCs for regenerative medicine-based applications. Such principles are based on their availability and ease of isolation using minimally invasive techniques from the pulpal tissues of extracted teeth, whilst mitigating many of the ethical issues associated with the collection of MSC populations from other tissue sources, coupled with their similarities to human bone marrow-derived MSCs (hBMMSCs), the current “gold standard” MSC source [2–4].

MSCs within both human exfoliated deciduous teeth (stem cells from human exfoliated deciduous teeth (SHEDs) [5]) and the permanent secondary dentition (human dental pulp stem/stromal cells (hDPSCs) [6, 7]) have been widely isolated and characterised, in terms of their distinct cell surface marker expression profiles, self-renewal and clonogenic characteristics, proliferative capacities, multipotent differentiation capabilities (e.g., dentinogenic, osteogenic, chondrogenic, adipogenic, myogenic, neurogenic, hepatogenic, and angiogenic lineages), and other desirable genotypic/phenotypic properties [8–12]. Therefore, hDPSCs, particularly those isolated from extracted third molar teeth due to orthodontic reasons, have received considerable attention for the development of more effective stem/stromal cell-based regenerative therapies. Indeed, despite much hDPSC-based research being dedicated toward demonstrating their beneficial effects as a regenerative dental pulpal therapy during endodontics [13, 14], hDPSCs have also been shown to promote tissue repair following transplantation into various animal model defects *in vivo*, related to pathologies associated with other clinical disciplines, such as orthopaedics, neurology, ophthalmology, hepatology, and cardiology [8, 11, 12, 15].

Despite such advances in our understanding of hDPSC biology and the continued development and evaluation of hDPSC-based therapies for clinical use, challenges remain which impact on the potential and exploitation of this MSC source in regenerative medicine. Although methodologies exist enabling the routine isolation of hDPSCs from dental pulp tissues, as with MSCs sourced from other tissues [16–19], an unequivocal issue which merits significant consideration is the established heterogeneous nature of MSC populations within the dental pulp, as isolated hDPSCs are invariably comprised of many individual subpopulations with contrasting biological and regenerative characteristics. Such traits have presented a major obstacle to the translational development of hDPSC-based therapies for clinical application, especially if hDPSC subpopulations possess divergent proliferation and differentiation properties to permit predictable and reproducible regenerative outcomes. Ever since the pioneering work of Gronthos et al. [6, 7], who originally described the characterisation of a unique population of postnatal hDPSCs from the dental pulp of human third molar teeth, the heterogeneity between hDPSC subpopulations within dental pulp tissues has been indisputable. Specifically, hDPSCs shared a similar immunophenotype to hBMMSCs and exhibited a high degree of clonogenicity, self-renewal, rapid proliferative rates, and multipotency capabilities, including differentiation into odontoblast-like cells and the production of sporadic, but densely calcified, nodules. Subcutaneous hDPSC transplantation into immunocompromised mice also resulted in the formation of a functional mineralised dentine-like tissue and associated dental pulp-like tissue *in vivo*, distinct to that formed by hBMMSCs [6, 7, 20]. However, further analysis of individual hDPSC subpopulations derived from single-cell colonies revealed significant differences in their proliferative and odontogenic potentials. Despite heterogeneous, multicolony hDPSC population expansion being capable of achieving >120 population doublings (PDs) *ex vivo*, only 20% of purified clonogenic hDPSC subpopulations were capable of proliferating >20PDs. Furthermore, only two-thirds of these hDPSC subpopulations were capable of forming abundant ectopic dentine *in vivo* [6–8].

Although Gronthos et al. concluded that isolated hDPSCs only represent a minor fraction of the total cell number within dental pulp tissues (approximately 400 fibroblastic colony-forming unit (CFU-F) colonies per 10^5^ cells plated) [6, 7], additional confirmation of the limited proportion of isolated hDPSC subpopulations capable of undergoing extensive *ex vivo* expansion and odontogenesis further highlighted the considerable heterogeneity surrounding the proliferative, lineage differentiation and other biological characteristics of hDPSC subpopulations. Thus, such circumstances have since confounded efforts to purify and profile large numbers of particular hDPSC subpopulations with the desired genotypic and phenotypic qualities required for the development of MSC-based therapies. As it has been estimated that undifferentiated MSC populations only comprise around 0.001-0.01% of the total number of cells within tissues such as adult human bone marrow [21], the procedures used for the harvesting of sufficient quantities of hDPSC subpopulations for evaluation and development for clinical use become a significant consideration. As a result of such low MSC yields from native tissues, extensive *ex vivo* expansion is often necessary to obtain sufficient cell numbers for successful therapy development, especially where allogenic MSC-based therapies are concerned [22, 23], with typically reported MSC therapeutic doses in the range of 10^8^ cells for routine cell transplantations [24, 25].

Consequently, a remaining challenge to the routine use of hDPSCs for regenerative medicine-based applications is the identification of particular molecular markers capable of discriminating hDPSCs with superior regenerative properties, versus lesser quality subpopulations. This review provides a comprehensive overview of our current understanding of hDPSC biology and the molecular basis behind hDPSC heterogeneity. Based on this knowledge, we further outline some of the key advances which have led to particular cellular markers being harnessed to distinguish between hDPSC subpopulations, in terms of their proliferative, multipotency, immunomodulatory, and other regenerative properties overall. Such characteristics may subsequently be exploited for the development of strategies that allow for the selective screening, improved isolation, and enrichment of superior quality hDPSC subpopulations from dental pulp tissues, leading to improved cell expansion, manufacture, and banking, thereby supporting the translational development of hDPSC-based therapy development.

## 2. Current Understanding of hDPSC Biology

### 2.1. Development and Stem Cell Niche Locations

**Figure 1:**
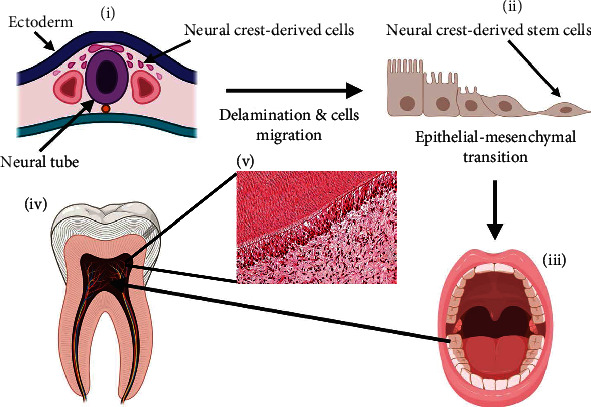
Summary of the events involved in hDPSC formation from migrating neural crest-derived cells during embryonic tooth development. (i) During development, neural crest-derived cells delaminate from the periphery of the neural tube and migrate to the oral region. (ii) Neural crest-derived cells undergo epithelial-mesenchymal transition, differentiating into neural crest stem cells and (iii) subsequently into several other cell types and tissues within the craniofacial region. (iv–v) These include the various cell types which comprise the dentine-pulp complex, including hDPSCs.

Despite dental pulp being recognised as a highly vascularised and innervated tissue comprising of a multi-heterogeneous population of cells [1], hDPSCs are established to be ectomesenchymal-derived stem cells, originating during embryonic tooth development from migrating cranial neural crest cells and possessing MSC-related properties [26–29]. During development, neural crest-derived cells delaminate from the periphery of the neural tube, migrate to the oral region, and undergo epithelial-mesenchymal transition, differentiating into neural crest stem cells and subsequently into several other cell types and tissues within the craniofacial region (Figure 1). As the self-renewal and multipotency of premigratory and postmigratory neural crest cells are thought to be maintained by neural crest-derived MSCs within developing tissues [30, 31], neural crest cells confer the advantageous regenerative properties of MSCs within the craniofacial region, including hDPSCs [11, 12, 27–33].

In postnatal tissues, hDPSCs remain quiescent within their stem cell niche microenvironments of the healthy dentine-pulp complex [34, 35], for instance, through their differentiation into newly formed odontoblast-like cells or restoration of pulpal fibroblast composition during tertiary (reparative) dentinogenesis [1, 32, 36–38]. Although the ontogeny, anatomical locations, and identities of hDPSCs within the dental pulp remain to be fully established, initial studies proposed that hDPSCs originate from within the cell-rich subodontoblast layer residing adjacent to the postmitotic primary odontoblasts, from fibroblast-like cell populations within the dental pulp stroma, and from perivascular regions associated with the pulpal vasculature [32, 37–41]. However, as hDPSCs adjacent to the primary odontoblasts are unlikely to contribute significantly to the regeneration of odontoblast-like cells during tertiary dentinogenesis [42], pericyte-derived subpopulations within the perivascular niche have since been established to possess particularly prominent roles in responding to tissue injury within the dentine-pulp complex, although contributions from nonpericyte-derived MSCs also occur [37–44]. Consequently, it is now believed that hDPSCs exist within several different niches throughout the dental pulp, albeit with distinct intrinsic characteristics and regenerative properties based on their respective locations within the tissue [3, 45, 46]. Thus, it has been speculated that the hDPSC subpopulations with contrasting developmental lineages within the dental pulp respond differently during tissue repair, which may account for the diverse proliferative and odontogenic responses originally observed within the dentine-pulp complex, following transplantation of individual single colony-derived hDPSC strains [1, 3, 6, 7].

### 2.2. Immunophenotypic Features

In accordance with the minimal criteria stipulated for the classification of human MSCs by the Mesenchymal and Tissue Stem Cell Committee of the International Society for Cell and Gene Therapy (ISCT) [47], hDPSCs exhibit adherence to tissue culture plastic under standard culture conditions and specific cell surface antigen expression (positive CD73, CD90, and CD105 expression and negative CD11b, CD14, CD19, CD34, CD45, CD79*α*, and human leukocyte antigen- [HLA]-DR expression) and exhibit multipotent differentiation capabilities of osteogenic, chondrogenic, and adipogenic lineages. However, although some controversy still surrounds the appropriateness and use of these specific criteria, particularly with contradictory reports around the expression of certain hematopoietic stem cell markers [48], it has been widely confirmed that hDPSCs demonstrate positive expression of the ISCT-recommended MSC markers, CD73 (5′-ectonucleotidase), CD90 (Thy-1), and CD105 (endoglin), and negative expression of the hematopoietic stem cell markers, CD3, CD8, CD11b, CD14, CD15, CD19, CD33, CD34, CD45, CD71, CD79*α*, CD117, and HLA-DR [6, 8, 9, 11, 12, 49–52]. However, there is currently no specific marker for hDPSCs, and although expression of a wide range of other mesenchymal, embryonic, neural crest, and other cell surface markers has been extensively examined, the heterogeneous nature of hDPSC subpopulations within dental pulp tissues and their distinct immunophenotypic characteristics have led to considerable inconsistencies and diversity being displayed in their marker expression profiles [9, 11, 48]. Nonetheless, in addition to CD73, CD90, and CD105, hDPSCs have been reported to express numerous other MSC surface markers, such as CD13 (aminopeptidase N), CD29 (*β*_1_-integrin), CD44, CD166 (activated-leucocyte cell adhesion molecule), and CD271 (low-affinity nerve growth factor receptor, LANGFR/p75) [9, 11, 49–56]. Consistent with their proposed location within the perivascular niche [37–44], hDPSCs have also been demonstrated to positively express perivascular cell (STRO-1 (stromal precursor antigen 1), STRO-3, and PDGFR-*β* (platelet-derived growth factor receptor-*β*)), endothelial cell (CD106, vascular cell adhesion molecule-1; CD146, melanoma cell adhesion molecule), smooth muscle cell (*α*-smooth muscle actin (*α*SMA)), and pericyte (3G5, ribosomal protein S14; NG2, neuron-glial antigen 2) markers, with hDPSCs predominantly presenting a pericyte-associated phenotype [6, 9, 12, 43, 44, 50–53, 56, 57].

Analysis of embryonic stem cell markers has revealed varying levels of OCT4 (octamer-binding transcription factor-4), NANOG (homeobox transcription factor), SOX2 (SRY- (sex determining region Y-) box 2), SSEA4 (stage-specific embryonic antigen-4), and Slug expression in hDPSCs, which regulate stem cell properties such as self-renewal, multi/pluripotency, and mesenchymal lineage commitment [9, 11, 49, 50, 53, 58–60]. Furthermore, hDPSCs exhibit positive gene expression for self-renewal and multipotency marker, BMI-1 [53, 61, 62]. Based on their neural crest origins, expression of various neural lineage markers has also been identified in hDPSCs, including CD117 (c-Kit), CD271, Nestin, glial fibrillary acidic protein (GFAP), *β*-III tubulin, S100, Notch 1, musashi-1, synaptophysin, microtubule-associated protein 2 (MAP-2), and oligodendrocyte-associated CNPase [11, 12, 49, 50, 52, 53, 63–65].

### 2.3. Self-Renewal and Multilineage Differentiation Characteristics

High self-renewal capabilities are one of the defining features of hDPSCs [2, 6–8]. Although hDPSCs and hBMMSCs both share similar spindle-shaped morphologies, gene expression profiles, and differentiation pathways overall, hDPSCs have been shown to maintain higher colony-forming efficiencies and proliferation rates than hBMMSCs, associated with the elevated expression of cell cycle-related genes, such as cyclin-dependent kinase 6 and insulin-like growth factor 2 (IGF-2), by hDPSCs [6–8, 66]. Indeed, heterogeneous hDPSC populations have been proven to possess considerable expansion potentials achieving >120PDs *ex vivo*, although considerable variations in the proliferative capacities of individual hDPSC subpopulations have been highlighted, as most are only capable of achieving <40PDs in culture [6–8, 52, 53, 67].

Under basal conditions, hDPSCs express osteogenic marker genes, including runt-related transcription factor 2 (RUNX2), type I collagen, dentine sialophosphoprotein (DSPP), osteocalcin, osteopontin, osteonectin, alkaline phosphatase, and bone morphogenetic proteins (BMP-2, BMP-4); adipogenic marker genes, such as peroxisome proliferator-activated receptor *γ* (PPAR*γ*), lipoprotein lipase (LPL), leptin, and adipophilin; chondrogenic markers, such as type II collagen and SOX9; and myogenic markers, such as *α*SMA, myosin, myogenin, and desmin [2, 6–8, 12, 49]. Such genotypic qualities support the extensive plasticity displayed by hDPSCs, a hallmark feature which makes these populations such attractive propositions in regenerative medicine, in terms of their potential to mature into more specialised cells for the potential repair of dental and nondental tissues throughout the body [11, 15, 27–29]. Under appropriate inductive conditions *in vitro*, hDPSCs can be induced to undergo differentiation into numerous cell types associated with both mesodermal and nonmesodermal (ectodermal and endodermal) lineages, including odontoblasts, osteoblasts, chondrocytes, adipocytes, glia cells, neuronal cells, oligodendrocytes, Schwann cells, retinal ganglion-like cells, endothelial cells, pancreatic cells, cardiomyocytes, hepatocytes, melanocytes, skeletal muscle cells, and bladder smooth muscle cells [8–12, 15, 50], well beyond the minimum multilineage differentiation criteria stipulated for hBMMSCs by the ISCT [47]. Such findings have subsequently led to further evaluation of hDPSC differentiation potency and regenerative potentials *in vivo*, most notably following transplantation into various animal disease and trauma models [8–12, 15].

### 2.4. Immunomodulatory and Anti-Inflammatory Properties

In addition to their proliferative and differentiation characteristics, hDPSCs have further been demonstrated to possess potent immunomodulatory and anti-inflammatory properties. hDPSCs do not express the major HLA class II surface antigen and are capable of inhibiting CD4^+^ and CD8^+^ T-cell proliferation and proinflammatory cytokine production, in addition to inducing their apoptosis. Such responses are induced by the secretion of soluble factors, such as human leukocyte antigen G5 (HLA-G5), interleukins (IL-6, IL-10), transforming growth factor-*β*_1_ (TGF-*β*_1_), hepatocyte growth factor (HGF), and Fas ligand (FasL), via hDPSC-derived exosome release and through the induction of endoplasmic reticulum (ER) stress in T-cells [68–75]. hDPSCs can also prevent T-helper 17 (Th17) cell activation, whilst stimulating regulatory T cell (Treg) differentiation, and suppress B cell proliferation and differentiation, influencing immunoglobulin production [69, 71–73, 76]. hDPSCs further attenuate activated peripheral blood mononuclear cell (PBMC) proliferation via TGF-*β*_1_, indoleamine 2,3-dioxygenase (IDO), and HGF secretion [77, 78] and regulate the differentiation and functions of macrophage subtypes through IDO-mediated inhibition of tumour necrosis factor-*α* (TNF-*α*) secretion and development of the proinflammatory M1 macrophage phenotype, in addition to stimulation of anti-inflammatory M2 macrophage polarisation by inhibiting Toll-like receptor (TLR) and nuclear factor *κΒ* (NF*κΒ*) signalling [78, 79].

## 3. Markers Implicated in Distinguishing hDPSC Subpopulations with Distinct Characteristics

**Figure 2:**
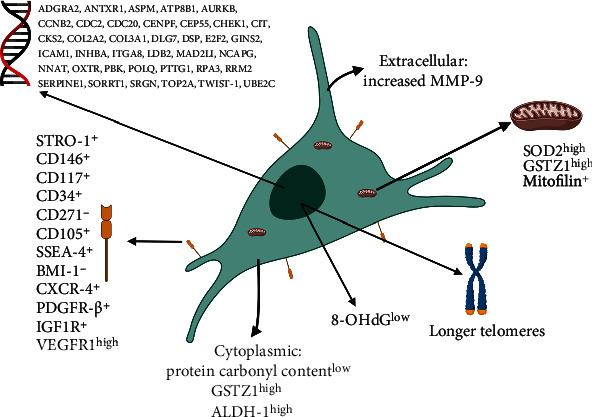
Summary of the subcellular locations of the proposed heterogenic gene, protein, and other biochemical markers implicated in distinguishing high stemness, proliferative, multipotency, and other regenerative characteristics between hDPSC subpopulations.

In light of the established heterogeneous nature of hDPSC subpopulations, a comprehensive understanding of the biological characteristics of hDPSCs has been crucial to efforts aimed at developing strategies for their exploitation as novel tissue regeneration therapies for clinical applications. Consequently, numerous studies have now reported the isolation and characterisation of single colony-derived clonal populations of hDPSCs utilising strategies and specific biological characteristics as markers, to selectively obtain more refined subpopulations for regenerative medicine purposes. A summary of these proposed heterogenic markers, their subcellular locations, and the purported hDPSC characteristics which these markers identify is presented in Figure 2 and Table 1.

### 3.1. Cell Surface Markers

To date, the most widely analysed and exploited biological characteristics described for the detection and purification of particular hDPSC subpopulations have been based on their relative expression of specific mesenchymal, embryonic, and neural crest cell surface markers [11, 50, 51]. Notably, early characterisation studies utilised a combination of perivascular markers (STRO-1, CD146, and 3G5), to colocalise STRO-1 and CD146 to the microvasculature and confirm that most hDPSCs reside within the perivascular niche of dental pulp tissues [43].

Numerous studies have since employed these and a variety of additional cell surface markers for the isolation and discrimination of distinct hDPSC subpopulations. STRO-1^+^/CD146^+^ subpopulations have been identified as highly proliferative, multipotent hDPSCs and are often coexpressed with other embryonic stem cell markers, such as OCT4 and NANOG, which aid the maintenance of MSC characteristics [59, 80]. These subpopulations have been shown to possess superior colony-forming efficiencies, compared to their STRO-1^−^/CD146^−^ counterparts, and able to proliferate >40PDs. STRO-1^+^/CD146^+^ hDPSCs are also capable of forming dentine/pulp-like structures [12], although certain STRO-1^+^/CD146^+^ clones were reported to exhibit restricted differentiation potential [80]. Thus, further attention has focussed on the characterisation of STRO-1^+^ hDPSC subpopulations also positive for hematopoietic stem cell markers, c-kit^+^ (CD117) and CD34^+^. These multipotent subpopulations can undergo osteogenic differentiation *in vitro* [81], whilst c-kit^+^/CD34^+^/STRO-1^+^ hDPSCs also coexpressing flk-1 (vascular endothelial growth factor receptor 2 (VEGFR2)) not only have strong osteogenic capacities but are also capable of angiogenic differentiation *in vitro*, codifferentiating into osteoprogenitor and endothelial progenitor cells [50, 82]. Furthermore, STRO-1^+^/c-Kit^+^/CD34^−^ and STRO-1^+^/c-Kit^+^/CD34^+^ hDPSCs have been proposed to represent distinct subpopulations, with contrasting cell proliferation, stemness, and differentiation properties, especially in terms of their ectodermal lineage capabilities, with STRO-1^+^/c-Kit^+^/CD34^+^ hDPSCs possessing a greater propensity towards neurogenic commitment [83]. It has since been shown that STRO-1^+^/c-Kit^+^/CD34^+^ hDPSCs, expressing CD271, Nestin, and SOX10, are capable of differentiating into Schwann cell-like cells *in vitro* and promoting axonal regeneration *in vivo* [84]. Thus, these studies suggest that a larger pool of hDPSCs exist within dental pulp tissues with enhanced multipotency towards mesodermal and ectodermal lineages, represented within a highly proliferative STRO-1^+^ population comprising several interrelated subpopulations [85].

Further studies into CD146^+^ hDPSCs have determined that these subpopulations are capable of promoting mineralisation and regenerating the dentine-pulp complex *in vivo*, identical to that formed by multicolony-derived hDPSCs [42, 86]. *In vivo* regenerated dentine and dentine-pulp complex were also significantly thicker and displayed uniform expression of dentine matrix protein-1 (DMP-1) and DSPP. Alternatively, CD146^−^ subfractions have been reported to exhibit potent neurogenic potential, with the additional elevated expression of neurotrophic factors [87].

As CD271^+^ cells are regarded as being of neural crest origin [63, 64], these have been identified as subpopulations with enhanced neurogenic potential, exhibiting high expression for neural markers, such as Nestin, SOX1, and SOX2, and the ability to differentiate into the neuronal cell lineage, compared to CD271^−^ hDPSCs [88]. Although positive CD271 expression is relatively low across all hDPSCs, highly proliferative, multipotent hDPSC subpopulations exhibit no CD271 expression (CD271^−^), unlike their low proliferative/unipotent CD271^+^ counterparts [52, 53]. In accordance with CD271 expression being proposed to significantly influence multipotent differentiation capabilities in hDPSCs, CD271^−^ subpopulations have been demonstrated to possess superior colony-forming efficiencies, prolonged proliferation, and multilineage potential *in vitro*, in addition to enhanced bone formation capabilities *in vivo* [64, 65]. Furthermore, CD271 has recently been identified to be highly expressed within STRO-1^+^/c-Kit^+^/CD34^+^ hDPSC subpopulations, possessing slow proliferation rates, reduced stemness, and early-onset senescence, compared to their STRO-1^+^/c-Kit^+^/CD34^−^ counterparts [83]. However, despite differences in CD271 expression, both hDPSC subpopulations exhibited similar osteogenic, myogenic, and adipogenic differentiation, although STRO-1^+^/c-Kit^+^/CD34^+^ hDPSCs expressing CD271 demonstrated greater neurogenic lineage commitment. That said, not all studies have demonstrated complete inhibition of multipotent differentiation in CD271^−^ expressing hDPSCs [63, 83].

Other MSC markers demonstrated to distinguish particular traits between hDPSC subpopulations include CD105, as CD105^+^ hDPSC subpopulations exhibit high proliferative, migratory, and multipotent differentiation potentials, especially towards the angiogenic lineages, exhibiting high expression of vascular endothelial growth factor (VEGF) and other proangiogenic factors such as granulocyte-macrophage colony-stimulating factor (GM-CSF) [89]. Upon transplantation into a mouse hind limb ischaemia model, CD105^+^ hDPSCs were able to regenerate high densities of capillaries within sites of injury. Such findings may be related to their ontogeny, as CD105 is a membrane glycoprotein expressed in vascular endothelium, as CD105^+^ cells are also found within the perivascular niche. Similar results were evident in a cerebral ischaemic model, whereby their transplantation resulted in new vessel formation in the ischaemic zone and subsequently promoted neuronal regeneration by endogenous neuronal cells [89].

CD51^+^/CD140*α*^+^ hDPSCs have also been identified as a subpopulation capable of odontogenesis, osteogenesis, and chondrogenesis. Their odontogenic and osteogenic differentiation capacity was demonstrated to be greater than that of the STRO-1^+^/CD146^+^ hDPSCs, producing greater quantified alkaline phosphatase activity and mineralised tissue formation [64]. However, despite the expression of other MSC markers, such as CD29^+^, CD44^+^, and CD73^+^, having been suggested to correlate with hDPSC stemness, these have been precluded as having potential benefits as markers for the isolation of hDPSC subpopulations [28].

Although the majority of isolated hDPSCs exhibit negative hematopoietic stem cell marker expression, a small percentage (≤2%) has been found to be positive for markers, such as CD34^+^ and CD117^+^ [8–12, 50, 51]. CD34^+^ hDPSC subpopulations have reduced proliferative capacities, but with an enhanced neurogenic potential, compared to their CD34^−^ counterparts [83, 84]. CD34^+^ clones also express lower levels of MSC markers, such as CD133 and CD44. CD34^+^ clones have the ability to undergo osteogenic, adipogenic, myogenic, and neurogenic differentiation. Most significantly, CD34^+^ hDPSCs show superior neurogenic potential and are capable of differentiating into Schwann cells, which upon *in vivo* transplantation were capable of sciatic nerve regeneration in an animal model [83, 84]. CD34^+^ hDPSC subpopulations express greater Nestin and CD271 than CD34^−^ clones and express GFAP. Subpopulations coexpressing STRO-1^+^/c-kit^+^/CD34^+^ were also able to undergo osteogenic, adipogenic, and myogenic differentiation *in vitro*, but no significant differences were evident in the differentiation capacities between CD34^−^ and CD34^+^ subpopulations [81, 83, 85]. CD117^+^ hDPSCs have been found to be capable of osteogenic, adipogenic, myogenic, and neurogenic differentiation, coexpressing with STRO-1^+^/CD34^+^ as described above [81, 83, 85]. CD117 (c-Kit) is a tyrosine kinase III receptor that acts in concert with stem cell factor (SCF) as its ligand, with proposed roles in maintaining the self-renewal properties of hDPSCs [90]. However, CD117 expression was gradually lost during differentiation [82].

In terms of embryonic stem cell and self-renewal markers, SSEA-4^+^ hDPSCs have been characterised as being highly proliferative subpopulations, with multipotent capacities towards osteogenic, chondrogenic, and neurogenic lineages, but impaired adipogenesis [58]. Furthermore, reduced BMI-1 expression has been correlated with the maintenance of stemness and extended proliferative properties in hDPSCs, albeit resulting in potential impairments in differentiation potential [61, 62]. Expression of the stromal cell-derived factor (SDF)-1*α* receptor and C-X-C chemokine receptor type 4 (CXCR-4, CD186) has further been identified to distinguish hDPSCs with greater colony formation efficiencies and proliferative and multilineage differentiation capacities than their CXCR4^−^ counterparts [57, 91, 92]. Similarly, hDPSCs sorted by their expression of PDGFR-*β* demonstrated that PDGFR-*β*^+^/c-kit^+^ subpopulations exhibited enhanced proliferation and prominent odontogenic differentiation *in vitro*, coupled with enhanced mineralisation and dentine/pulp-like tissue formation *in vivo* [93]. IGF1 receptor (IGF1R), regarded as a pluripotent marker of embryonic stem cells, was also found to be expressed in hDPSCs, with IGF1R^+^ subpopulations displaying both self-renewal and multipotency potentials, especially towards neurogenic and angiogenic lineages [94]. Similarly, enriched populations of VEGFR1^high^ hDPSCs have a strong ability to undergo angiogenic differentiation *in vitro*, producing increased blood vessel sprouting and neovascularisation than VEGFR1^low^ subpopulations [95].

### 3.2. Markers Related to Self-Renewal, Proliferation, and Resistance to Cellular Senescence

In light of the integral importance of self-renewal, clonogenicity, colony-forming efficiency, and *ex vivo* expansion potential to the development of hDPSCs for regenerative medicine, numerous studies have established a number of relevant cellular markers capable of distinguishing hDPSC subpopulations with superior self-renewal and proliferative capabilities. As detailed above, STRO-1^+^/CD146^+^, CD271^−^, BMI-1^−^, and CXCR-4^+^ hDPSC subpopulations have been shown to possess superior colony-forming efficiencies and stemness properties than their STRO-1^−^/CD146^−^, CD271^+^, BMI-1^+^, and CXCR4^−^ counterparts [57, 59, 62, 64, 65, 80, 83, 91, 92].

Another significant drawback of hDPSC heterogeneity stems from the original findings that only 20% of purified clonogenic hDPSC subpopulations are capable of undergoing >20PDs [6–8], prior to proliferative decline and the onset of replicative (telomere-dependent) senescence. Such events significantly impede the *ex vivo* expansion capabilities of hDPSCs necessary to produce sufficient cell numbers for clinical use, characterised by progressive telomere shortening, inhibition of G_1_-S phase transition, and permanent growth arrest. This is associated with the loss of telomeric TTAGGG repeats, positive senescence-associated *β*-galactosidase staining, and increased tumour suppressor (p53 and retinoblastoma protein (pRb)) and cyclin-dependent kinase inhibitor (p21^waf1^ and p16^INK4a^) gene expression [96, 97]. These events are recognised to significantly alter the MSC genotype and phenotype, ultimately leading to impaired cellular regenerative properties and disrupted local tissue microenvironment signalling mechanisms, through the secretome associated with the senescence-associated secretory phenotype (SASP) [96–98].

Although hDPSC susceptibility to replicative (telomere-dependent) and oxidative stress-induced (telomere-independent) premature senescence has previously been recognised [52, 53, 61, 62, 99], the relative expression levels of many cell surface markers have been implicated as being indicative of elevated rates of hDPSC proliferation and/or expansion potential, including STRO-1^+^, CD34^−^, CD90^+^, CD105^+^, CD117^+^, CD146^+^, CD271^−^, CXCR4^+^, PDGFR-*β*^+^/c-kit^+^, and IGF1R^+^ [50, 57, 59, 64, 65, 80–83, 85, 86, 89, 91–94]. Reduced BMI-1 expression in hDPSCs has been demonstrated to delay replicative senescence and limit senescence marker (positive senescence-associated *β*-galactosidase staining and elevated p16^INK4a^ expression) detection [62].

Additional studies have focussed on understanding the telomere dynamics underlying hDPSC heterogeneity and the intrinsic mechanisms responsible for protecting highly proliferative hDPSC subpopulations from accelerated telomere erosion. SSEA-4^+^ hDPSCs have been found to possess longer telomeres and higher proliferation rates, compared to SSEA-4^−^ subpopulations [58]. More recently, significant variations in the *ex vivo* expansion capabilities of individual hDPSC subpopulations have been demonstrated, with highly proliferative hDPSCs capable of achieving >80PDs, whereas low proliferating hDPSCs only complete <40PDs before senescence, correlating with hDPSCs with high proliferative capacities possessing longer telomeres. This leads to the delayed detection of senescence-related markers, such as positive senescence-associated *β*-galactosidase staining and elevated p53, p16^INK4a^, and p21^waf1^ expression [52]. Thus, it is likely that such highly proliferative hDPSCs are responsible for the extensive expansion potential of heterogeneous hDPSC populations (>120PDs) *in vitro*, as previously described [6–8]. Low proliferative hDPSC senescence was also associated with the loss of stem cell marker characteristics, positive CD271 expression, and impaired osteogenic/chondrogenic differentiation, in favour of adipogenesis. In contrast, highly proliferative hDPSCs exhibited no CD271 expression but retained stemness and multipotency capabilities, only demonstrating impaired differentiation following prolonged *in vitro* expansion (>60PDs). As most studies have only reported negligible reverse transcriptase human telomerase catalytic subunit (hTERT) expression in hDPSCs [52, 53, 61, 100, 101], hTERT is unlikely to be responsible for maintaining telomere integrity and the proliferative/multipotency capabilities of highly proliferative hDPSCs. Thus, the absence of hTERT implies that other intrinsic mechanisms may account for differences in telomere lengths, proliferation rates, and differentiation capabilities between high and low proliferative hDPSC subpopulations.

Oxidative stress is another prominent mediator of cellular senescence in MSCs, associated with the excessive generation of reactive oxygen species (ROS) at the expense of endogenous enzymic and nonenzymic antioxidant defence mechanisms, leading to indiscriminate oxidative damage to biomolecules, such as DNA, proteins, and lipids and accelerating premature senescence [53, 96, 97, 102]. As with previous studies confirming differences in replicative senescence susceptibilities between high and low proliferative hDPSCs [52], similar variations in the relative susceptibilities of hDPSC subpopulations to premature senescence have also been confirmed, following continual exposure to oxidative stress [53]. Although all hDPSC subpopulations exhibit accelerated susceptibilities to premature senescence, highly proliferative hDPSCs (CD271^−^) showed most resistance to premature senescence, achieving 50-76PDs similar to untreated controls (>80PDs). In contrast, low proliferative subpopulations (CD271^+^) collectively displayed accelerated premature senescence (4-32PDs), even in untreated controls. Whilst telomere lengths were largely unaffected by oxidative stress exposure, elevated premature senescence susceptibilities in low proliferative hDPSCs (2-10PDs) were accompanied by the loss of certain stem cell markers and increased oxidative DNA (8-hydroxy-deoxy-guanosine (8-OHdG)) and protein (protein carbonyl content) damage, absent in highly proliferative hDPSCs until 45-60PDs [53]. Such findings of enhanced low proliferative hDPSC subpopulation susceptibilities to oxidative damage are supported by recent single-cell Raman spectroscopy studies, which demonstrated distinctive decreases in nucleic acid and protein spectral intensities in low proliferative hDPSCs, as a consequence of accumulative exposure to ROS-induced biomolecular damage [103].

Further studies led to the discovery that increased superoxide dismutase 2 (SOD2) and glutathione S-transferase *ζ*1 (GSTZ1) expression and SOD activities were present in highly proliferative hDPSCs (10-25PDs), which declined during culture expansion [53]. However, low proliferative hDPSCs (2-10PDs) exhibited inferior SOD-, catalase-, and glutathione-related antioxidant expression and activities overall. As mitochondria are the principle cellular source of ROS during senescence, mitochondrial SOD2 and mitochondrial/cytosolic GSTZ1 are likely candidates as the principle protective enzymic antioxidant defence mechanisms against oxidative stress in highly proliferative hDPSC subpopulations, preventing mitochondrial damage and hDPSC senescence and leading to the extended maintenance of proliferative, stemness, multipotency, and other cellular characteristics [52, 53, 62, 96, 104–107]. Thus, such telomere length, senescence, oxidative stress, and antioxidant characteristics may be utilised as predictors of hDPSC proliferative and multipotency qualities for future regenerative medicine exploitation [108]. Evidence is increasingly emerging to highlight mitochondrial dynamics, metabolism, oxidative stress, and function as having a major impact on the phenotypic responses of hDPSCs and other MSC populations, such as differentiation [109–111]. Indeed, cell proliferation-inducing protein 52 (mitofilin) is an antagonist of mitochondrial activation during differentiation located within the inner mitochondrial membranes of hDPSCs, which becomes depleted during normal differentiation [112]. Consequently, the selective isolation of mitofilin^+^ hDPSCs has been shown to result in the isolation of more primitive cells with greater differentiation efficiencies. Therefore, a better understanding of the molecular profiles, mitochondrial-related stem cell markers, and morphological characteristics of hDPSC mitochondria may further prove effective in the selection of superior quality hDPSC for clinical applications.

### 3.3. Multipotency or Specialised Differentiation Markers

Another essential facet of hDPSC stemness, which makes them appealing options for regenerative medicine, is their potential multipotency capabilities [11, 15, 27–29]. Consequently, a wide range of studies have implicated individual or collections of cell surface markers as being indicative of multipotent differentiation characteristics in hDPSC subpopulations, including STRO-1^+^/CD146^+^[59, 80], c-kit^+^/CD34^+^/STRO-1^+^ [50, 81–83], CD51^+^/CD140*α*^+^ [64], CD105^+^ [89], CD271^−^ [52, 53, 64, 65, 83], CXCR4^+^[57, 91, 92], and IGF1R^+^ [94]. Similarly, SSEA-4^+^ hDPSCs possess multipotent capacities towards osteogenic, chondrogenic, and neurogenic lineages but impaired adipogenesis [58]. However, reduced BMI-1 expression is associated with limited hDPSC differentiation potential [61, 62]. Furthermore, despite not distinguishing multipotent properties, certain cell surface markers have been attributed to the identification of hDPSC subpopulations with specific mesodermal or ectodermal differentiation lineage capabilities. For instance, STRO-1^+^/c-Kit^+^/CD34^+^, CD146^−^, and CD271^+^ hDPSCs have a greater propensity towards neurogenic commitment [83, 84, 87, 88], whilst CD105^+^, VEGFR1^high^, and PDGFR-*β*^+^/c-kit^+^ subpopulations have strong tendencies for angiogenic [89, 95] and odontogenic [93] lineage differentiation, respectively. Thus, such limited lineage differentiation tendencies may point towards more refined indications for such subpopulations in future for more specific regenerative applications, such as nerve injury and cardiovascular or tooth repair, thereby utilising these hDPSCs for optimised clinical scenarios for which they appear best suited.

Multipotency has further been revealed to be influenced by hDPSC telomere lengths and relative susceptibilities to replicative senescence, with hDPSC subpopulations with longer telomere lengths and higher proliferation rates displaying tripotent osteogenic, chondrogenic, and adipogenic lineage differentiation, in contrast to low telomere length, unipotent hDPSCs which only exhibit adipogenesis [52, 58]. As high and low telomere length hDPSCs express CD271^−^ and CD271^+^ levels, respectively [52, 53], positive CD271 expression in low telomere length hDPSC subpopulations may explain their more lineage-restricted capabilities, considering the established inhibitory effects of CD271 on multipotent differentiation in hDPSCs [64, 65].

### 3.4. Immunomodulatory Markers

Of the various hDPSC characteristics which may be exploited for regenerative medicine purposes, the abilities of specific markers to selectively distinguish hDPSC subpopulations with exceptional immunomodulatory and anti-inflammatory potencies are the area which is currently most limited [11, 68–79]. However, IGF1R^+^ hDPSCs have been reported to possess immunomodulatory and anti-inflammatory properties, following *in vivo* transplantation into a rodent hypoxia-ischemia model [94].

### 3.5. Other Markers Associated with Other Regenerative Characteristics

hDPSC subpopulations expressing the intracellular enzyme, aldehyde dehydrogenase-1 (ALDH-1), associated with improved stemness have been found to be capable of osteogenic, chondrogenic, and adipogenic differentiation. These subpopulations have also been immunolocalised to the perivascular niche and the perineurium of nerve bundles [113]. The relative abilities of hDPSC subpopulations have further been exploited through their contrasting migratory responses to GM-CSF in culture, with highly migratory hDPSCs also exhibiting improved proliferative responses and other regenerative properties overall [114, 115]. Although most studies have established hDPSC heterogeneity using two-dimensional (2D) culture approaches, a recent study demonstrated that highly proliferative/multipotent and low proliferative/unipotent hDPSCs in three-dimensional (3D) type I collagen gels exhibit comparable gel contraction capabilities and matrix metalloproteinase-2 (MMP-2) expression/activities, although highly proliferative/multipotent hDPSC subpopulations possess higher MMP-9 expression/activities, which may impact on the abilities of these subpopulations to regulate cellular functions within the stem cell niche and remodel/degrade 3D biomaterial scaffolds; and their regenerative properties overall [116].

Microarray studies by Menicanin et al. [117] compared the global gene expression profiles of highly proliferative/multipotent hDPSC clones with low proliferative potential cell clones with restricted differentiation potential, in order to identify potential biomarkers of highly proliferative subpopulations with multipotent capabilities. In total, 24 genes were identified to be upregulated in highly proliferative/multipotent hDPSCs associated with cell cycle progression, mitosis, and cell division; DNA repair and replication; gene transcription; and cell proliferation and differentiation (Table 2). A more recent study by Kobayashi et al. [80] reported similar findings, revealing 1227 genes that were related to multipotency, 90 of which were also associated with stemness or differentiation. Based on their relative levels of expression, 14 of these 90 genes were selected as candidate hDPSC markers, particularly in relation to their multipotency, stemness, or differentiation characteristics, between high proliferative/multipotent hDPSCs and low proliferative/lineage-restricted hDPSC subpopulations (Table 3). Thus, the characterisation of such hDPSC subpopulations provided an array of novel candidate marker genes of increased stemness, proliferation, and multipotency, facilitating their improved isolation and the enrichment of superior quality hDPSCs for regenerative medicine applications.

## 4. Future Perspectives and Considerations

Although individual hDPSC subpopulations do share certain similarities, hDPSC heterogeneity is now a well-established concept. Furthermore, despite a greater insight into the complex molecular factors and processes that underpin hDPSC heterogeneity being achieved since their original characterisation by Gronthos et al. [6, 7] and advances in the identification of a plethora of purported markers that discriminate between distinct hDPSC subpopulations, many important questions remain to be addressed. Firstly, why do diverse niches of hDPSC subpopulations exist within the dentine-pulp complex with contrasting immunophenotypes and proliferative/differentiation potentials, despite these tissues primarily consisting of odontoblasts and pulpal fibroblasts [1, 3, 6, 7, 32, 39, 40, 118]. Certain hDPSC subpopulations would undoubtedly be responsible for replenishing odontoblasts and pulpal fibroblasts lost due to disease and trauma during tertiary dentinogenesis [1, 32, 36–44, 119]. However, considering the neural crest origins of ectomesenchymal-derived hDPSCs, together with the highly vascularised and innervated nature of dental pulp tissues [1, 26–32], it is reasonable to assume that other hDPSC subpopulations are responsible for vascular and neural cell replacement and the identification of neural and perivascular cell markers within the dental pulp [6, 9, 11, 12, 37–44, 49–53, 56, 57, 63–65]. That said, as the identification of stem cell markers is an essential prerequisite to enable the selective screening, isolation, and purification of hDPSC subpopulations for particular therapeutic applications, it remains plausible that additional minor hDPSC subpopulations exist within dental pulp tissues which are yet to be isolated and explored, due to a lack of understanding regarding their intrinsic stem cell marker properties, niche locations, and roles within the dentine-pulp complex.

Although the impact of underlying factors associated with the individual patient donors of the permanent dentition used as sources of hDPSCs, such as their genomic composition, age, gender, health, diet, and environmental and other unknown factors, on the genotypic and phenotypic characteristics of hDPSC subpopulations is still open to speculation and remains to be fully established, these are highly likely to contribute to the issue of hDPSC heterogeneity [19]. However, from a wider perspective, more pertinent questions which certainly provide significant contributions to the complex heterogeneous nature of hDPSCs surround the developmental origins, hierarchical organisation, and precise niche locations of individual subpopulations within dental pulp tissues, in addition to the extent to which the entire heterogeneous populations within the dental pulp are comprised of true multipotent hDPSCs or a collection of unique committed progenitor cells with specialised lineage-restricted differentiation capabilities [9, 80]. Furthermore, despite standard methods being established for the isolation and characterisation of hDPSCs [120], the methods of derivation, cell culture conditions, the stage of the cell cycle, and proliferation or commitment upon isolation again could be influential factors on the regenerative properties of hDPSCs and warrant additional consideration [19, 120].

### 4.1. Developmental Origins

Gronthos et al. [6, 7] originally proposed that the heterogeneous nature of hDPSCs may reflect differences in their developmental stages or may even represent different pulpal cell lineages. Since then, studies into the developmental potentials of different hDPSC clones have suggested that a number of subpopulations exist within the dental pulp, derived from either the mesoderm or ectoderm of migratory cranial neural crest cell origins [26–29, 64, 83]. Several studies indicate that intrinsic positional information can dictate the neural crest stem cell phenotype within tissues and that environmental signals can regulate neural crest cell developmental fate and differentiation. As postmigratory neural crest cells only comprise a small proportion of the larger DPSC population overall and their multipotency is believed to persist within tissues [27–33], it is plausible that such differential developmental origins within the DPSC population contributes to their heterogenic nature. However, pericyte-derived subpopulations within the perivascular niche have been ascribed principal roles in mediating tissue repair responses within the dentine-pulp [37–44]. It has also been proposed that the relative contribution of pericyte-derived and nonpericyte-derived MSCs to cell differentiation in tissues depends on the extent of the vascularity and its kinetics of growth and/or repair. Thus, in tissues with high vascularity, such as dental pulp, the pericyte contribution to MSCs would be expected to be considerable [1, 121].

Although details on the nature and developmental origins of individual hDPSC subpopulations within human dental pulpal tissues have largely remained elusive, much progress has been made through the study of a mouse incisor stem cell model, regarded as an attractive system for the study of adult dental stem cell biology [122]. This model has permitted investigations into the properties, distinct locations, and contributions of active MSC subpopulations to the constant growth and repair of dentine and pulp tissues within continuously erupting incisor teeth, to compensate for tissue loss during occlusion. These constantly active MSCs can be subsequently distinguished from lesser active MSCs resident within the molar teeth, which do not undergo continual growth in adult mice [32, 118]. It has been established that incisor MSCs are a heterogeneous population, consisting of cells from different neural crest-derived tissues, with the ectomesenchymal cells giving rise to dental pulp and odontoblast cells, as evident in humans. Furthermore, through exploitation of genetic-based lineage tracing, it was revealed that differentiated odontoblasts originate from perivascular NG2^+^ pericytes during mouse incisor growth [44, 123]. It has further been shown that all NG2^+^ perivascular cells are derived from GLI family zinc finger 1 (Gli1^+^) cells, which are preferentially localised surrounding the vasculature. Although the majority of Gli1^+^ cells in the mouse incisor do not express classic MSC markers, such as CD44, CD73, CD105, CD146, or Nestin, Gli1^+^ cells are activated in response to incisor injury. Thus, Gli1^+^ cells are the major source for odontoblasts and pulp cells during incisor growth and repair, although in contrast to incisors, mouse molars do not contain Gli1^+^ cells around the dental pulp vasculature, whereas NG2^+^ pericytes are present [124]. However, as lineage tracing quantification indicated that only 15-16% of newly differentiated odontoblasts were derived from NG2^+^ perivascular cells, other MSC-like cells of nonpericyte origin were also shown to be present in the dental pulp and contribute to the majority of odontoblasts. Indeed, as certain MSCs have been shown to differentiate from peripheral nerve-associated glial cells [125], lineage tracing of Schwann cells as the predominant glial cell type confirmed that odontoblast-like cells originate from neural crest-derived Schwann cells and Schwann cell precursors, thereby initiating reparative dentinogenesis and supporting dental pulp cells through the formation of Schwann cell-derived odontoblasts. Therefore, as Schwann cells do not express perivascular markers, pericytes and Schwann cells are regarded as distinct dental stem cell populations within the neurovascular bundle regions of mouse incisor dental pulp, with diverse contributions to homeostasis and repair.

Lineage tracing experiments have since shown that *α*SMA-expressing, perivascular niche-derived MSCs generate a small number of newly formed odontoblasts during primary dentinogenesis, although their contribution to the formation of new odontoblast-like cells during reparative dentinogenesis is far more significant [126]. Further studies have also identified that 30% of MSCs in continually growing mouse incisors exhibit positive CD90 expression during postnatal development, although CD90^+^ MSCs decrease in number during adulthood [127]. However, following adult incisor injury, CD90^+^ MSCs reappear and contribute to reparative processes, replenished by mitotic cells within the mouse incisor pulp, positive for hematopoietic stem cell marker, Celsr1^+^. Such lineage tracing analysis has also discovered Axin2^+^ cells in the odontoblast layer and the dental pulp in the proximal region of the mouse incisor, whose progeny contributes to dental pulp cell and odontoblast numbers, implying that Axin2^+^ cells are transit amplifying cells (TACs) [127]. Similarly, Axin2^+^ cells in mouse molars differentiate into new odontoblast-like cells that secrete the reparative dentine via Wnt/*β*-catenin signalling in response to injury [128]. Additionally, PDGFR*β*^+^ cells are recognised as identifying MSCs within the cervical loop region and TACs of the mouse incisor model [129], which are distinct MSC populations to those found in the neurovascular niches [123–125]. Therefore, markers, such as NG2^+^, Gli1^+^, CD90^+^, *α*SMA^+^, Celsr1^+^, Axin2^+^, and PDGFR*β*^+^, amongst others yet to be identified, may shed light on similar hDPSC subpopulations within the perivascular niches of human dental pulp tissues and their roles in repair and regeneration within the dentine-pulp complex.

### 4.2. Stem Cell Niches within the Dental Pulp

It is widely accepted that MSCs reside in quiescent states within various specialised niches and uniquely organised local microenvironments that enable the maintenance and regulation of MSC self-renewal, proliferation, migration, and differentiation in response to injury, via direct cell-cell/cell-matrix interactions and communications mediated through secreted factors [6, 130, 131]. From original suggestions that hDPSCs originate from niches within the cell-rich subodontoblast layer, the dental pulp stroma, and especially from perivascular regions surrounding the pulpal vasculature [32, 37–44], it is now believed that several stem cell niches exist within the dental pulp containing distinct multipotent hDPSCs and other regenerative characteristics, supported by the identification of hDPSC subpopulations of pericyte and nonpericyte origins during previous human and mouse localisation and lineage tracing studies, as described above. However, further research is still necessary to explore the precise locations of as yet unexplored stem cell niches within the dental pulp and the extent to which local niche microenvironments influence hDPSC heterogeneity. Not only would such undertakings help in understanding whether isolated multipotent hDPSCs are derived from one highly proliferative multipotent population or from many committed hDPSC progenitor subpopulations with distinct lineages, but also assist in endeavours to develop more novel 3D scaffold materials which recapitulate the physiochemical properties of the native stem cell niche microenvironment, leading to improved regulation of transplanted hDPSC regenerative responses *in situ* [132–134].

### 4.3. Hierarchical Organisation

The ability to self-renew is one of the defining features of hDPSCs, recognised as involving either the slow cell division of an original mother stem cell to generate daughter cells with identical developmental potential to the original mother stem cell during symmetric division; or mother stem cell division into an identical copy of the mother cell and a highly proliferative TAC, possessing multipotent differentiation capabilities during asymmetric division. However, as TACs further divide to form larger colonies, they achieve a more mature progeny with reduced proliferative capabilities and the induction of replicative senescence, becoming more lineage-restricted. Consequently, stem cells expanded during development are maintained in quiescent states within the homeostatic stem cell niche microenvironments and participate in tissue repair as required, upon exposure to tissue perturbations or stressors [135, 136]. TACs have been suggested to arise within the postnatal dental pulp in response to cavity-induced injury and first to differentiate into new odontoblast-like cells [37].

As with MSCs derived from other tissues, the presence of a hierarchy amongst hDPSCs in adult dental pulp, with a small subpopulation of self-renewing, highly proliferative multipotent stem cells resident within a larger compartment of predominantly less proliferative and more committed, bipotent or unipotent subpopulations, has been proposed for some time [6, 7, 137]. Nonetheless, the findings of more recent reports on hDPSC heterogeneity would certainly support the hierarchical model, in that small minority subpopulations within the dental pulp are highly proliferative, multipotent hDPSCs, whilst the majority are low proliferative, more lineage-restricted bipotent or unipotent hDPSCs [52, 53]. These highly proliferative, multipotent hDPSCs are believed to be responsible for maintaining the stem cell pool through their self-renewal ability and differentiation into cells of different lineages [1, 6]. As highly proliferative, multipotent hDPSCs with longer telomeres are expanded *ex vivo* and become senescent, they lose their proliferative capabilities and also become more lineage-restricted with bipotent or unipotent properties [52, 53]. As asymmetric stem cell division involves true mother cells giving rise to a multitude of differentiated daughter cells without themselves going through a high number of cell divisions, only a limited amount of telomere shortening occurs in such cells, thereby maintaining telomere length integrity [138]. Therefore, the characterisation of individual highly proliferative/multipotent and low proliferative/unipotent hDPSC subpopulations with contrasting telomere length profiles conceivably lends credence to the existence of a hierarchical arrangement amongst hDPSCs residing within the dental pulp.

Differential gene expression profiles between highly proliferative/multipotent hDPSC subpopulations and their low proliferative, bipotent or unipotent counterparts may also reflect these being different entities within the hierarchical organisation, as these genes associated with key responses, such as cell cycle progression, mitosis, and cell division; DNA repair and replication; cell proliferation; stemness; and differentiation, were commonly identified to be upregulated in highly proliferative/multipotent hDPSCs, which would expectedly contribute to the superior retention of stemness, proliferative, and differentiation characteristics versus their low proliferative, bipotent/unipotent counterparts (Tables 2 and 3) [80, 117]. Thus, hDPSC heterogeneity can be determined via distinct gene expression and the functionality and frequency of cell cycle transitions.

Nucleoside labelling and lineage tracing studies using the mouse incisor stem cell model [32, 118, 122] have exploited the differences between quiescent/slow-cycling (label-retaining) cells and fast-cycling cells (TACs), to investigate the tissue locations of rapidly cycling and slow-cycling cells. Using this approach, TACs expressing genes associated with polycomb repressive complex 1 (Prc1), such as Ring1a (Ring1) and Ring1b (Rnf2), have been confirmed as the most rapidly cycling cells in the mouse incisor dental pulp, located immediately distal to slow-cycling MSCs and crucial for mediating the TAC phenotype via Wnt/*β*-catenin signalling [139]. Furthermore, the Gli1^+^ cells located within neurovascular bundles which provide the vast majority of odontoblasts and pulp cells during incisor growth and repair have been reported to colocalise with slow-cycling cells within the dental pulp [124]. CD90^+^ slow-cycling cells have been elucidated to be DPSCs which contribute to odontoblasts and pulp cells throughout the life of the mouse incisor and responsible for incisor growth [125]. However, CD90^+^ DPSCs only contribute to a small proportion of odontoblasts and pulp cells, correlating with the proportion of CD90^+^ slow-cycling cells. Additional studies have found that around half of pulp cells and odontoblasts in the mouse incisor model were glial-derived, located within the population of slow-cycling cells with nonglial-derived, pericyte populations possibly contributing to the remainder [125]. Therefore, current evidence suggests that fast-cycling cells account for cell replenishment to maintain tissue homeostasis, whereas quiescent/slow-cycling cells act as a “reservoir” to be initiated to supply TACs, upon tissue injury [127, 140]. However, in contrast to hDPSC biology as a whole, much less emphasis has been directed towards understanding their TACs, despite their integral roles in repair and regenerative responses within the dentine-pulp complex.

### 4.4. Isolation, Purification, Characterisation, and Culture

Protocols for the routine isolation, purification, characterisation, and culture of hDPSCs are well-documented [9, 11, 19, 120, 141–143]. However, although the diverse range of methodologies reported for the harvesting of hDPSCs from dental pulp tissue achieve such aims overall, these do not overcome some of the current challenges which remain, in terms of addressing issues surrounding the consistent isolation and enrichment of hDPSCs with enhanced stemness, proliferative, and multipotent differentiation characteristics; in particular, as such hDPSCs are regarded as minor populations in the dental pulpal milieu [1, 6, 7, 52, 53, 137]. Thus, the development of novel strategies to permit the standard screening, collection, and expansion of particularly high-potency hDPSC subpopulations from dental pulp tissues would certainly be the key to meeting existing inadequacies relating to hDPSC heterogeneity.

Technical advances are being made, with most exploiting the molecular characteristics associated with hDPSCs with enhanced stemness, proliferative, and multipotent differentiation properties, as described above. Of these, the differential mesenchymal, embryonic, and neural crest cell surface marker profiles reported between hDPSC subpopulations have been most widely exploited to date, particularly using antibody- and molecular biology-based techniques, such as fluorescence-activated cell sorting (FACS), magnetic-activated cell sorting (MACS), or real-time quantitative reverse transcription polymerase chain reaction (qRT-PCR) [11, 51, 52, 55, 56]. However, despite the multitude of cell surface markers identified, uncertainty remains whether such markers are specifically associated with the undifferentiated progenitor state of hDPSCs or even if these markers are actually MSC specific, or whether particular markers are more useful in identifying distinct hDPSC subpopulations individually or with other coexpressed markers. That said, even studies involving multiple stem cell markers have proven challenging, as it is difficult to distinguish whether hDPSC subpopulations are mutually exclusive of each other and if the characteristics of subpopulations that express a certain cell surface marker differ to other subpopulations that coexpress the same marker with additional surface markers. Furthermore, as most studies share a common limitation of having a small sample size of donors from which the hDPSCs were harvested, it remains to be established whether hDPSC molecular profiles are representative across a wider population or a consequence of donor-donor variation. Alternative initiatives to address some of the concerns regarding the use of cell surface markers have included the identification of new cell surface proteome markers in hDPSCs isolated from single donors, using label-free mass spectrometry [56]. Of the 101 CD markers and 286 non-CD cell surface markers, these included TNF receptor superfamily proteins (CD40, CD120a, CD261, CD262, CD264, and CD266), integrins (*α*-4, *α*-6, and *α*-10), and IL receptors (CD121a, CD130, CD213a1, CD217, and CDw210b), which could be utilised for the more precise identification and isolation of hDPSCs. Similarly, multiparametric flow cytometry has recently been reported to permit the detection of several cell surface molecules, with which to characterise and identify the phenotypes of heterogeneous hDPSC subpopulations, both *in vitro* and *in vivo* [144]. The cell surface plasma membrane of hDPSCs has further been the focus of innovative studies aimed at enriching, separating, and identifying putative membrane protein markers by mass spectrometry, such as CD9, CD10 (neprilysin), and CD63, a novel approach which may be utilised for the characterisation and profiling of hDPSC subpopulations in future [145].

Gene expression profile comparisons of highly proliferative/multipotent hDPSC versus low proliferative/lineage-restricted hDPSCs have led to the discovery of potential genotypic marker genes for the selective isolation and purification of highly multipotent hDPSCs for regenerative medicine applications (Tables 2 and 3) [80, 117]. In contrast to these ground-breaking microarray studies, proteomic profiling comparisons of highly proliferative/multipotent hDPSC versus low proliferative/lineage-restricted hDPSCs have been much less in comparison [146, 147]. However, in studies involving low numbers of clones obtained from multiple donors, gene expression and proteomic differences amongst clones isolated from multiple donors may not truly reflect genotypic and phenotypic differences between highly proliferative/multipotent and low proliferative/lineage-restricted hDPSCs, but the genetic backgrounds of the donors instead [80]. Consequently, gene expression and proteomic analyses should be performed with patient-matched, highly proliferative/multipotent hDPSCs and low proliferative/lineage-restricted hDPSCs isolated from individual donors, thereby eliminating the influences of donor variation.

An alternative and greater understanding of hDPSC subpopulations within the dental pulp may be obtained utilising single-cell RNA sequencing (scRNA-seq), which allows the transcriptomic profiling of thousands of individual cells and is widely applied in stem cell biology for the analysis of MSC heterogeneity and the provision of specific markers by cell clustering, predicting cell fate by making trajectories, understanding the difference or dysregulation between different cell types, stage, or status, and providing indications for lineage tracing studies [148, 149]. Such sophisticated approaches can be expanded further through the combination of DNA, RNA, protein, and/or the epigenomic analyses, to permit the high-dimensional dissection of single cells, which offers great potential for understanding the regulation of molecular pathways. scRNA-seq, coupled with lineage tracing studies, has now been performed using mouse models, which have further highlighted the diverse nature of DPSCs within the developing tooth [150]. Additional reports utilising scRNA-seq have confirmed the high level of MSC heterogeneity within the dental pulp complex, with the existence of an active pool of DPSCs responsible for the formation of the principle mesenchymal-derived cell types, odontoblasts and distal/apical dental pulp cells, which produce a continuum of transient cell states [151]. The apical region of the dental pulp also contains active progenitor-like cell and precursor stromal-like cell subpopulations. Such findings were further validated in human wisdom teeth, which continue to grow until later in life. In comparison of molar hDPSC subpopulations to DPSCs within the mouse incisor dental pulp, the hDPSCs within the nongrowing human tooth preferentially exhibited a transcriptional state of more mature cells associated with the distal pulp, whilst those in the growing human tooth possessed a more apical-like transcriptomic profile [151]. Thus, it appears that nongrowing teeth are particularly characterised by a default distal pulp-like state, whilst an apical-like state is a signature of growing dental tissues. The presence of DPSC subpopulations and quiescent/active cell populations within the apical tooth region are supported by analogous scRNA-seq studies involving the mouse incisor model, which have identified a subpopulation of Runx2^+^/Gli1^+^ cells within the heterogeneous Gli1^+^ population [152]. These Runx2^+^/Gli1^+^ cells are not MSCs in nature but are located in close proximity to MSCs and TACs, where they maintain the MSC niche and regulate TAC functions, via IGF signalling. These studies have further confirmed the occurrence of a novel Foxd1^+^ DPSC subpopulation in the apical region near the epithelial labial cervical loop, where these are capable of differentiating towards odontogenic or pulpal lineages [150, 151].

As highly proliferative/multipotent hDPSCs are minority subpopulations within dental pulp tissues, the development of noninvasive strategies capable of successfully discriminating between hDPSC subpopulations with contrasting proliferative and differentiation capabilities *in situ* would be immensely beneficial for the selective screening and isolation of more desirable hDPSCs for *in vitro* assessment and therapy development. Consequently, single-cell Raman spectroscopy signatures obtained for highly proliferative/multipotent and low proliferative/lineage-restricted hDPSCs have been proven to be a viable noninvasive tool for the rapid screening and isolation of superior quality hDPSCs from dental pulp tissues *in situ*, thereby overcoming issues surrounding hDPSC heterogeneity [103].

## 5. Implications of Advances in Our Understanding of hDPSC Heterogeneity

It is undeniable that our understanding of the molecular basis underlying hDPSC heterogeneity has improved significantly in recent years. Although hDPSC heterogeneity has hindered their development for clinical application, the need for a greater understanding of their molecular characteristics has led to the identification of a wide variety of novel cell surface, gene, protein, and metabolic markers, purported to reliably discriminate between hDPSC subpopulations with contrasting stemness, proliferative, multipotency, immunomodulatory, anti-inflammatory, and other relevant regenerative properties. Importantly, considering that hDPSCs only constitute a minor fraction of the total cell content within dental pulp overall, the identification and subsequent isolation and enrichment of highly proliferative, multipotent hDPSCs as even smaller minority subpopulations within the dental pulp become a much greater challenge. In light of the extensive *ex vivo* expansion required to obtain sufficient cell numbers for successful MSC-based therapy development [22, 23], highly proliferative hDPSC subpopulations with extended proliferative lifespans that retain stemness and multipotency capabilities could be regarded as ideal candidates to progress towards *ex vivo* laboratory evaluation and translational development as regenerative medicine-based therapies for broad clinical applications. That said, it has been suggested that lesser proliferative hDPSC subpopulations may be better suited in the development of more specialised tissues, in line with their restricted differentiation potentials down certain lineages, thereby expediting their possible tailoring towards more specific regenerative purposes.

Through the identification of new potential markers which distinguish hDPSC subpopulations with specific or superior characteristics, these will undoubtedly lead to advancements in the development of novel screening, isolation, and purification strategies, permitting the routine and effective identification, enrichment, and expansion of specific hDPSC subpopulations from whole dental pulp tissues for regenerative medicine applications. However, despite these recent advancements, many important aspects of hDPSC biology remain unanswered, which significantly impacts on their development as cellular therapeutics. For instance, despite cell surface proteins being extensively regarded as viable markers to distinguish hDPSC subpopulations, limitations in their specificity highlight the requirement for the identification of further markers, especially those capable of differentiating between highly proliferative/multipotent and low proliferative/lineage-restricted hDPSCs. As numerous markers were identified during gene expression profiling studies [80, 117], more extensive studies into the profiles of highly proliferative/multipotent and low proliferative/lineage-restricted hDPSC subpopulations using various genomic and proteomic technologies warrant further investigation. However, such studies would benefit from the inclusion of larger cohorts of patient donors, to confirm the most reliable gene, protein, or metabolic markers identified and discount the influences of donor genetic variations.

The reasons behind hDPSC heterogeneity remain to be fully established, although the developmental origins, hierarchical organisation, and stem cell niche locations of hDPSC subpopulations are strong contributory factors to the ultimate question as to what extent heterogeneous hDPSC populations within the dental pulp are derived from true multipotent hDPSCs or many different committed cell subpopulations exhibiting more specialised lineage-restricted differentiation capabilities [9, 80]. Nonetheless, existence of the hierarchical model is supported by recent reports that small minority subpopulations within the human dental pulp are highly proliferative, multipotent hDPSCs, whilst the majority are low proliferative, more lineage-restricted bipotent or unipotent hDPSCs [6, 7, 52, 53, 137, 138], further supported by the discovery of rapidly cycling TACs and slow-cycling cells relevant to the hierarchical structure, within the mouse incisor stem cell model [32, 44, 118, 122–129, 139, 140]. Such studies have further confirmed the prominent presence of DPSC subpopulations within both perivascular and neural niche locations associated with the neurovascular bundles of mouse incisor dental pulp, with contrasting roles in tissue homeostasis and repair [32, 44, 118, 122–129, 139, 140], helping to corroborate the presence of pericyte-derived hDPSC subpopulations within the perivascular niche of human dental pulp [37–44].

It is unquestionable that the development of transgenic mouse models to study stem cell incisor repair, coupled with technological advances in lineage tracing and scRNA-seq [32, 44, 118, 122–129, 139, 140, 148–152], has aided insights into DPSC subpopulation heterogeneity within the mouse incisor model, through the identification of specific cell types, status, and functions. An existing drawback of these techniques is the broad expression levels of currently used gene markers between neural crest cell and DPSC subpopulations. Additionally, as most lineage tracing experiments of dental pulp cells have solely been performed using the mouse incisor repair model, a key question remains over the relative applicability of such studies to nongrowing molar teeth in mouse models, in addition to whether comparative findings would be evident within human incisors and molars. Indeed, species variations in the cell subtypes and transcriptional profiles involved in tooth self-renewal have recently been highlighted between continuously erupting mouse incisor and nonerupting molars, in addition to growing and mature teeth in humans [151]. However, other recent reports have utilised scRNA-seq technologies to map the transcriptional landscape of the various cell populations that comprise human teeth, including the hDPSCs and other cell types within the dental pulp and their niche microenvironments [153]. By utilising such approaches, hDPSCs were characterised by their higher expression of Frizzled-related protein (FRZB), Notch receptor 3 (NOTCH3), CD90 (THY1), and smooth muscle myosin heavy chain 11 (MYH11), in line with their MSC and perivascular nature. Additionally, despite previous ethical concerns surrounding the possible use of lineage tracing experimentation in humans, recent developments have also demonstrated that it is now possible to trace human cell lineages using natural variations in nuclear/mitochondrial DNA and in DNA methylation status [154, 155]. Thus, although further characterisation studies into the diversity of neural crest cell and mouse DPSC subpopulations *in vivo* are warranted, such combined lineage tracing and scRNA-seq analyses of hDPSCs in human dental pulp tissues could allow us to finally address the remaining issues restricting the translational development of hDPSCs for future clinical use, through a better understanding of the cellular and molecular mechanisms regulating tooth development, homeostasis, and tissue repair, relevant to improved regenerative therapies in the future.

## 6. Final Conclusions

It is inevitable that hDPSC heterogeneity has posed major hurdles to their translational development and evaluations in clinical trials. Indeed, it is recognised that only a limited number of hDPSC-based clinical trials have occurred to date, due to concerns regarding the optimisation of isolation and culture expansion protocols, safety, mechanisms of action, good manufacturing practice (GMP), and quality control procedures and regulations [16, 19, 120, 156, 157]. Therefore, by addressing these remaining issues and harnessing their specific properties overall, the utilisation of specific markers for the discrimination of more desirable highly proliferative/multipotent hDPSC subpopulations could become a routine strategy for their selective isolation and purification in the future. Such innovative measures would ultimately aid their overall expansion, assessment and efficient hDPSC manufacture, cryopreservation, and banking [9, 12, 16, 18, 156, 158], thereby supporting the successful translational development of more effective hDPSC-based regenerative therapies for a wide range of potential dental and nondental clinical applications.

## Figures and Tables

**Table 1:** Summary of the cell surface, senescence and other relevant gene, protein and metabolic markers implicated in distinguishing high stemness, proliferative, multi-potency and other regenerative characteristics between hDPSC sub-populations.

Marker	Associated properties of hDPSC sub-populations	References
STRO-1^+^	High colony forming efficiency, high proliferation, multi-potent	[59, 80, 83, 85]
CD146^+^	High colony forming efficiency, high proliferation, multi-potent, high odontogenic differentiation	[43, 59, 80, 86]
CD146^−^	High neurogenic differentiation	[87]
CD117^+^ (c-kit^+^)	High odontogenic, osteogenic, neurogenic, adipogenic, myogenic and angiogenic differentiation	[50, 81–84, 94]
CD34^+^	Low stemness, low proliferation, high osteogenic, neurogenic and angiogenic differentiation	[50, 81–84]
CD271^+^	Low proliferation, bi-/uni-potent, high neurogenic differentiation	[52, 53, 88]
CD271^−^	High colony forming efficiency, high proliferation, multi-potent	[52, 53, 64, 65, 83]
CD105^+^	High proliferation, high migration, multi-potent, high angiogenic differentiation	[89]
CD51^+^ (CD140*α*^+^)	High odontogenic, osteogenic and chondrogenic differentiation	[64]
SSEA-4^+^	High proliferation, multi-potent, high osteogenic, chondrogenic and neurogenic differentiation, low adipogenic differentiation	[58]
BMI-1^−^	High stemness, high proliferation, low multi-potency	[61, 62]
CXCR-4^+^ (CD186)	High colony forming efficiency, high proliferation, multi-potent	[57, 91, 92]
PDGFR-*β*^+^	High proliferation, high odontogenic differentiation	[93]
IGF1R^+^	High stemness, high proliferation, multi-potent, immunomodulatory, anti-inflammatory	[94]
VEGFR1^high^	High angiogenic differentiation	[95]
Long Telomeres	High proliferation, resistance to senescence, high stemness, multi-potent	[53, 103]
Low Oxidative DNA & Protein Biomarkers	High proliferation, resistance to senescence, high stemness, multi-potent	[53, 104]
SOD2^high^	High proliferation, resistance to senescence, high stemness, multi-potent	[52, 53]
GSTZ1^high^	High proliferation, resistance to senescence, high stemness, multi-potent	[52, 53]
Mitofilin^+^	High stemness, multi-potent	[112]
ALDH-1^high^	High stemness, multi-potent	[113]
MMP-9^high^	High stemness, high proliferation, multi-potent, increased matrix remodelling	[116]

**Table 2:** Summary of the marker genes identified to exhibit upregulated expression by microarray analysis, which distinguishes between high proliferative/multipotent hDPSCs and low proliferative hDPSC subpopulations. Adapted from Menicanin et al. [117].

Gene functions	Gene symbol
Cell cycle, mitosis, and cell division	ASPM, AURKB, CCNB2, CDC2, CDC20, CENPF, CEP55, CIT, CKS2, DLG7, MAD2L1, NCAPG, PBK, PTTG1, UBE2C
DNA repair and replication	CHEK1, E2F2, GINS2, POLQ, PTTG1, RPA3, RRM2, TOP2A
Transcription regulation	CENPF, E2F2, LDB2, PTTG1, TWIST-1
Cell proliferation and differentiation	CENPF, CHEK1, CIT, CKS2, TWIST-1

**Table 3:** Summary of the marker genes relating to multipotency, stemness, or differentiation, identified to be differentially expressed by microarray analysis, which distinguishes between high proliferative/multipotent hDPSCs and low proliferative/lineage-restricted hDPSC subpopulations. Adapted from Kobayashi et al. [80].

	Gene symbol
Genes positively correlated with multipotency	ATP8B1, DSP, ICAM1, INHBA, NNAT, OXTR, SERPINE1, SORT1, SRGN
Genes negatively correlated with multipotency	ADGRA2, ANTXR1, COL1A2, COL3A1, ITGA8

## Data Availability

No additional data were available to support this review article.

## Abbreviations

2D: Two-dimensional
3D: Three-dimensional
8-OHdG: 8-Hydroxy-deoxy-guanosine
*α*-SMA: *α*-Smooth muscle actin
ALDH-1: Aldehyde dehydrogenase-1
BMP: Bone morphogenetic protein
CFU-F: Fibroblastic colony-forming unit
CXCR-4: C-X-C chemokine receptor type 4
DMP-1: Dentine matrix protein-1
DSPP: Dentin sialophosphoprotein
ER: Endoplasmic reticulum
FACS: Fluorescence-activated cell sorting
FasL: Fas ligand
FRZB: Frizzled-related protein
GFAP: Glial fibrillary acidic protein
Gli1: GLI family zinc finger 1
GM-CSF: Granulocyte-macrophage colony-stimulating factor
GMP: Good manufacturing practice
GSTZ1: Glutathione S-transferase *ζ*1
hDPSC: Human dental pulp stem/stromal cell
HGF: Hepatocyte growth factor
HLA: Human leukocyte antigen
hTERT: Human telomerase catalytic subunit
IDO: Indoleamine 2,3-dioxygenase
IGF1R: IGF1 receptor
IGF-2: Insulin-like growth factor 2
IL: Interleukin
ISCT: International Society for Cell and Gene Therapy
LANGFR: Low-affinity nerve growth factor receptor
LPL: Lipoprotein lipase
MAbs: Monoclonal antibodies
MACS: Magnetic-activated cell sorting
MAP-2: Microtubule-associated protein 2
MHC: Major histocompatibility complex
MMP: Matrix metalloproteinase
MSC: Mesenchymal stem/stromal cell
MYH11: Smooth muscle myosin heavy chain 11
NF*κΒ*: Nuclear factor *κΒ*
NG2: Neuron-glial antigen 2
NOTCH3: Notch receptor 3
OCT4: Octamer-binding transcription factor-4
PDGFR-*β*: Platelet-derived growth factor receptor-*β*
PDs: Population doublings
PPAR*γ*: Peroxisome proliferator-activated receptor *γ*
pRb: Retinoblastoma protein
Prc1: Polycomb repressive complex 1
qRT-PCR: Quantitative reverse transcription polymerase chain reaction
ROS: Reactive oxygen species
RUNX2: Runt-related transcription factor 2
SASP: Senescence-associated secretory phenotype
SCF: Stem cell factor
scRNA-seq: Single-cell RNA sequencing
SDF-1*α*: Stromal cell-derived factor-1*α*
SOX: SRY- (sex-determining region Y-) box
SOD2: Superoxide dismutase 2
SSEA-4: Stage-specific embryonic antigen-4
STRO: Stromal precursor antigen
TAC: Transit-amplifying cell
TGF-*β*_1_: Transforming growth factor-*β*_1_
Th17: T-helper 17
TLR: Toll-like receptor
TNF-*α*: Tumour necrosis factor-*α*
VEGF: Vascular endothelial growth factor
VEGFR: Vascular endothelial growth factor receptor.
